# An Investigation of the Post-laryngectomy Swallow Using Videofluoroscopy and Fiberoptic Endoscopic Evaluation of Swallowing (FEES)

**DOI:** 10.1007/s00455-017-9862-7

**Published:** 2018-01-19

**Authors:** Margaret M. Coffey, Neil Tolley, David Howard, Michael Drinnan, Mary Hickson

**Affiliations:** 1 Imperial College Healthcare Trust, SLT Department, Charing Cross Hospital, Ground Floor, South Wing, Fulham Palace Road, London, W6 8RF UK; 2 Imperial College Healthcare Trust, ENT Department, St Mary’s Hospital, Praed Street, London, W2 1NY UK; 3 Imperial College Healthcare Trust, ENT Department, Charing Cross Hospital, Fulham Palace Road, London, W6 8QX UK; 4 Regional Medical Physics Dept, Freeman Hospital, Freeman Road, Newcastle upon Tyne, NE7 7DN UK; 5 Institute of Health and Community, Plymouth University, Derriford Road, Plymouth, Devon PL6 8BH UK; 6 Department of Surgery and Cancer, Imperial College London, London, UK

**Keywords:** Laryngectomy, Dysphagia, FEES, Videofluoroscopy

## Abstract

This study investigates the post-laryngectomy swallow. Presence and degree of residue on the post-laryngectomy swallow as observed on videofluoroscopy and FEES is described. In addition, videofluoroscopy and FEES are assessed for reliability and inter-instrument agreement. 30 laryngectomy subjects underwent dysphagia evaluation using simultaneous videofluoroscopy and FEES. These were reviewed post-examination by three expert raters using a rating scale designed for this purpose. Raters were blinded to subject details, type of laryngectomy surgery, pairing of FEES and videofluoroscopy examinations and the scores of other raters. There was a finding of residue in 78% of videofluoroscopy ratings, and 83% of FEES ratings. Comparison of the tools indicated poor inter-rater reliability and poor inter-instrument agreement. Dysphagia is an issue post laryngectomy as measured by patient self-report and by instrumental evaluation. However, alternative dysphagia rating tools and dysphagia evaluation tools are required to enable accurate identification and intervention for underlying swallow physiology post laryngectomy.

**Electronic supplementary material:**

The online version of this article (10.1007/s00455-017-9862-7) contains supplementary material, which is available to authorized users.

## Introduction

Laryngectomy surgery involves the anatomical separation of respiratory and swallowing systems. In contrast with other dysphagic populations, the risk of aspiration is low in this group, occurring only in the event of fistualisation or voice prosthesis leakage. Nonetheless, dysphagia is increasingly recognised [1–4] as a significant problem post laryngectomy. Some of the pathophysiological issues which may compromise swallowing ability post laryngectomy include pseudodiverticulum [4] [5, 6], fistualisation [4, 6–8], stricture [4, 9–11], fibrosis [12, 13], impaired pharyngeal propulsion [14], voice prosthesis leakage, [15–18] and reflux [19, 20]. These difficulties may lead to impaired delayed bolus transit, bolus obstruction and sometimes bolus regurgitation. Difficulties with dysphagia post laryngectomy may result in prolonged mealtimes, compromised nutrition and weight loss [21], [3] decreased psychological wellbeing and distress [2] and diet and social interaction limitations [1]. However, in contrast to other dysphagic populations, there remains limited data on the presentation of dysphagia or the best evaluation tool to facilitate optimum management.

## Instrumental Assessment of Swallowing

### Videofluoroscopy (VF)

Videofluoroscopy allows radiographic examination of the dynamic swallow process [22] and has traditionally been considered the gold standard for dysphagia evaluation [23].

A limited number of X-ray imaging studies have investigated dysphagia in the post laryngectomy patient [24–27]. Videofluoroscopy has also been combined with manometry (Videomanofluorography) to examine dysphagia post laryngectomy [5], [28] [14].

### Fibreoptic Endoscopic Evaluation of Swallow (FEES)

FEES involves passing a flexible endoscope through the nose and towards the pharynx to observe swallowing in real time. FEES is a reliable and sensitive tool for assessing dysphagia [29]; given accessibility to patients and avoidance of X-ray exposure it has challenged the predominance of VF in the clinical setting.

FEES has been used extensively to evaluate swallowing in the head and neck cancer population, [30–36], and aspects of communication following laryngectomy [37–40]. However, the use of FEES to evaluate swallow post laryngectomy has not been reported.

### Simultaneous Comparison of VF and FEES

Dysphagia can vary greatly between patients, but also from one swallow to the next in the same patient. In an instrumental comparison, the best experimental design is to evaluate the instruments on the same subject to eliminate inter-subject variability, and at the same time to eliminate intra-subject variability.

In the majority of studies [40–45] videofluoroscopy and FEES were carried out consecutively in the same patients. Performing videofluoroscopy and FEES evaluations simultaneously is technically challenging and has been described in a limited number of studies [46–49].

To date, all simultaneous and consecutive studies of videofluoroscopy and FEES have been undertaken in subjects with a larynx. This study is the first investigation of simultaneous FEES and videofluoroscopy to evaluate dysphagia in post laryngectomy patients.

## AIMS

AIM 1: To describe the presence of swallow residue post-laryngectomy.

AIM 2: To describe the degree of swallow residue post laryngectomy.

AIM 3: To assess the reliability and inter instrument agreement of the two principal tools for dysphagia management; videofluoroscopy (VF) and fibre-optic endoscopic evaluation of swallowing (FEES).

## Methods

Ethical approval was granted by London Riverside Research Ethics Committee (Reference number: 10/H0706/25).

### Participants

A convenience sample of eligible patients were recruited from the outpatient surveillance caseload of a large head and neck cancer centre in the UK. We excluded participants who:

- Did not have a voice prosthesis;
- Were less than 3 months post-surgery or completion of postoperative oncological treatment;
- Had documented cognitive dysfunction;
- Were unable to tolerate placement of a flexible nasendoscope.

### Simultaneous Swallow Assessment

Each subject’s swallowing was examined using simultaneous videofluoroscopy and FEES.

### Videofluoroscopy

The fluoroscopy unit GE Medical Systems Model UIH40CCD JK (GE, Amersham, UK) was used to capture images at a rate of 30 frames per second onto a Sony DVD recorder DVO 1000MD, (Sony, Weybridge, UK).

### Fibre-Optic Endoscopic Evaluation of Swallowing

A Pentax FNL10RBS flexible nasendoscope (Pentax New Jersey, USA) was passed through right nares and advanced from the velopharyngeal port, past the base of tongue to the level of the voice prosthesis. If the subject experienced discomfort when the scope was passed through the right nares, the scope was removed and passed through the left nares. FEES exams were recorded onto the Kay Pentax Swallow Work Station Model 7127e (Pentax New Jersey, USA).

### Swallow Boluses

Each subject had 4 trial swallows in each of four consistencies. These were:

• Thin liquid (L): 10 ml of Gastrografin radio opaque contrast (Bayer PLC, Newbury UK) with 0.5 ml Silver Spoon green food colouring, (British Sugar PLC).

• Puree (P): 10 ml of Ambrosia Devon custard (Premier foods, St Albans UK) with barium (made from 150 ml of custard mixed with 3 tablespoons of E-Z-HD barium sulfate powder 98% w/w (Bracco UK Ltd, High Wycombe, UK),

• Soft solid (S): 1 cm thick slice of a medium yellow banana smeared with 3 ml of custard and barium mix, as described above.

• Hard solid (H): ¼ digestive biscuit smeared with 3 ml barium custard mix.

### Swallow Bolus Imaging

The following swallows were recorded using simultaneous VF and FEES. First, the subject was positioned in the lateral oblique plane to allow a clear view of the voice prosthesis under VF. Three trials of each consistency were given, the bolus being recorded in transit from the oral cavity to the upper esophagus. After the three trials, the subject took a water rinse swallow before moving to the next consistency.

It was considered important to observe swallows in both planes in order to screen all stages of swallowing, including the esophageal phase. Therefore, following all trials in the lateral oblique position, the subject was placed in the antero-posterior plane with the nasendoscope remaining in place. The subject then completed one further trial of each consistency, the bolus being recorded from oral cavity to esophagus. After each trial in the antero-posterior plane, the subject took a water rinse swallow.

For clarity, the order of swallows and water rinses was as follows:

Lateral-oblique: L1 L2 L3 rinse P1 P2 P3 rinse S1 S2 S3 rinse H1 H2 H3 rinse.

Antero-posterior: L4 rinse P4 rinse S4 rinse H4 rinse.

## Expert Rating of Swallows

### Swallow Rating Scale

As there was no suitable scale available for the evaluation of swallowing residue post laryngectomy, a 24-point consensus derived scale (Electronic supplementary material 1) was developed for rating of VF and FEES swallow evaluations in laryngectomy patients. Face and content validity of the scale was established through discussion and consultation with experienced members of a head and neck cancer multidisciplinary team. Additionally, laryngectomy patients provided input about the crucial aspects of their swallow difficulty and opinions on what should be included on this rating scale. The scale assessed the presence and degree of residue in the following anatomical regions of interest: neopharynx, voice prosthesis and upper esophagus. Presence of residue was indicated by answering the question “Is there residue on/in (voice prosthesis/neopharynx/esophagus) on (thin, puree, soft, solid) using a binary yes/no tick box scale. Degree of residue was measured on a visual analogue scale anchored by minimal (00 mm) and severe (100 mm).

Three expert raters were recruited, each with at least 5 years’ experience in a large Head and Neck cancer centre where they manage laryngectomy patients daily. Each rater underwent 2 days of group training to maximise reliability and confirm that the rating scale was suitable for use with both videofluoroscopy and FEES.

### Expert Rater Evaluation

Considering first the VF images, the recorded dynamic swallows from each patient were presented to the three raters. Participants were presented in random order, with the individual swallows segmented for each participant according to consistency described in the methods. Raters could review each swallow exam as many times as needed.

The raters scored the swallow sequence for each consistency (i.e. 3 Lateral Oblique + 1 Antero-Posterior swallows) using the swallow rating scale. The entire exercise was repeated for the FEES images, with the patients in a different random order so that raters could not link examinations from the different tools. Raters evaluated videos for both videofluoroscopy and FEES examinations without audio recording to reduce recall bias.

## Statistical Analysis

Data was entered and analysed in IBM SPSS version 23 (IBM Armonk, New York). Visualisation was performed in Microsoft Excel.

### AIM 1: Presence of Swallow Residue Post Laryngectomy

Here we describe the overall pattern of residue, for each anatomical region of interest and bolus type, and for all anatomical regions of interest and bolus types combined, according to the expert raters. Since we used two instrumental assessments and cannot claim that either is a definitive (gold standard) measure, we report the data separately for VF and for FEES.

As ratings related to presence of residue yielded categorical data, a consensus score for three raters was calculated from the ratings of each clinician. Consensus score was calculated when two or more raters agreed.

Agreement was then investigated between FEES and VF. A contingency table was arranged quoting the number of positive responses. Data was then analysed using McNemars to assess the differences between videofluoroscopy and FEES.

### AIM 2: Degree of Swallow Residue Post Laryngectomy

As ratings related to degree of residue yielded continuous data, the difference between both FEES and Videofluoroscopy as measured in millimetres on the visual analogue scale was plotted against the mean score for each subject to produce a Bland–Altman plot, see electronic supplementary information 2. In calculating the difference between videofluoroscopy and FEES, videofluoroscopy was subtracted from FEES, therefore a positive mean difference represents a higher score from FEES, whereas a negative mean difference represents a higher score from videofluoroscopy. A *t* test was undertaken to assess significance.

### AIM 3: Reliability and Inter Instrument Agreement Using VF and FEES

This is one of few studies to report simultaneous VF and FEES outcomes, and the only study to report these data in the post-laryngectomy swallow. If we are to use our tools reliably, then it is important to understand the agreement within and between tools.

### Inter-rater Reliability

Reliability between raters was assessed by comparing the three expert assessments of each swallow sequence, for each anatomical region of interest. Reliability for Videofluoroscopy and FEES was investigated using free marginal kappa for categorical data. Free marginal Kappa was chosen because raters were not forced to assign a certain number of cases to each category and therefore had free rather than fixed marginals. In addition, as this study involved more than two raters, the multirater free marginal Kappa was used to examine both intra and inter rater reliability for categorical data. Intraclass Correlation Coefficient (ICC). was used to examine intra and inter rater reliability for continuous data.

Inter instrument agreement was analysed using Fleiss kappa.

## Results

A complete set of images was obtained for 30 subjects; two subjects were excluded due to failure of endoscopy recording equipment. Demographic characteristics are outlined in Table 1.

**Table 1:** Demographic characteristics

Age	66.3 (SD 8.6) years range 43–81 years
Time since surgery	89.9 (SD 63.3) months range 4–225 months
Gender	
Female	6 (20%)
Male	24 (80%)
Ethnicity	
Black/black british	1 (3%)
White	26 (87%)
Asian/asian british	3 (10) %
Tumour type	
T1	1 (3%)
T2	4 (13%)
T3	7 (23%)
T4	11 (37%)
Unknown	7 (23%)
Surgery	
Total laryngectomy	22 (73%)
Pectoralis major flap	3 (10%)
Radial forearm flap	1 (3%)
Jejunum flap	3 (10%)
Jejunum and pectoralis major flap	1 (3%)
Myotomy	
Yes	24 (80%)
Not applicable	3 (10%)
Unknown	3 (10%)
Radiotherapy Hx	
None	3 (10%)
Pre-operative XRT	13 (43%)
Postoperative XRT	12 (40%)
Pre & postoperative XRT	2 (7%)
Chemotherapy Hx	
Pre op chemo	5 (17%)
No chemo	25 (83%)
Salvage surgery	
Yes	17 (57%)
No	13 (43%)

### AIM 1: Presence of Residue in the Post-laryngectomy Swallow

Table 2 shows the results relating to presence of residue in anatomical regions of interest with different consistencies. This data came from rating scale categorical questions “Is there residue on/in the (voice prosthesis/neopharynx/esophagus) on (thin liquids/puree/soft/solid) and represents the percentage of positive responses for each tool. The raters systematically found it much easier to identify residue in the neopharynx using videofluoroscopy compared to FEES whatever the consistency. For residue on the voice prosthesis there was little difference between the tools, except for puree. For esophageal residue FEES was different to videofluoroscopy on solid consistency only.

**Table 2:** Differences between tools—presence of residue

Parameter	Videofluoroscopy		FEES		*P* < 0.001
	Consistency	%	Consistency	%	*P*
Percentage of positive responses for presence of neopharynx residue	Thin liquids	100% 24/30	Thin liquids	23.3% 0/0	0.001* N/A
	Puree	83.3% 25/29	Puree	6.6% 0/0	0.001* N/A
	Soft	86.6% 20/28	Soft	13.3% 2/2	0.001* 1.0
	Solid	80% 24/30	Solid	6.6% 0/0	0.001* N/A
Percentage of positive responses for presence of voice prosthesis residue	Thin liquids	73.3% 22/30	Thin liquids	80% 25/28	0.18 0.5
	Puree	90% 27/30	Puree	0% 27/27	0.001* 0.3
	Soft	80% 24/30	Soft	93% 25/26	0.22 0.4
	Solid	66.6% 21/30	Solid	93.3% 27/27	0.39 0.008
Percentage of positive responses for presence of upper esophageal residue	Thin liquids	90% 27/30	Thin liquids	93.3% 26/28	1.0 1.0
	Puree	96.6% 29/30	Puree	93.3% 27/27	1.0 1.0
	Soft	80% 24/30	Soft	93.3% 24/26	0.75 0.3
	Solid	66.6% 20/30	Solid	96.6% 29/29	0.001* 0.002

Missing values removed. Proportions are expressed as number positive/number rated

### AIM 2: Degree of Residue

Videofluoroscopy scored a greater degree of neopharyngeal residue on all consistencies, see Table 3. The degree of voice prosthesis residue was similar for both tools on all consistencies except for thin liquids when FEES scored a greater degree of residue. Both tools showed a similar degree of esophageal residue for puree and soft consistencies. However FEES scored a greater degree of esophageal residue on thin liquids and solids. While each of these differences were statistically significant it is noted that limits of agreement between tools are wide.

**Table 3:** Differences between tools—degree of residue

Parameter	Mean difference* (95 CI)		*t* –test *P* value < 0.05	Limits of agreement (mm)
Degree of neopharynx residue	Thin liquids *N* = 1	− 10.98 (− 16.90, − 5.05) N/A	0.001 N/A	− 42.09 LL to 20.12 UL N/A
	Puree *N* = 1	− 20.11 (− 28.67, − 11.55) N/A	0.001 N/A	− 65.03 LL to 24.81 UL N/A
	Soft *N* = 2	− 14.55 (− 23.74, − 5.36) + 20.5 (60.34) − 521.5 to + 562.6	0.003 0.7	− 62.79 LL to 33.69 UL − 97.8 to + 138.8
	Solid *N* = 2	− 19.44 (− 29.72, − 9.17) + 35.1 (42.78) − 349.3 to + 419.4	0.001 0.5	34.48 LL to 73.36 UL − 48.7 to + 118.9
Degree of voice prosthesis residue	Thin liquids *N* = 28	22.03 (13.93, 30.12) + 27.0 (24.48) + 17.5 to + 36.5	0.001 < 0.001	− 20.26 LL to 64.42 UL − 21.0 to + 75.0
	Puree *N* = 29	0.72 (− 7.51, − 8.95) + 3.8 (25.12) − 5.80 to + 13.31	0.859 0.4	42.48 LL to 43.93 UL − 45.4 to + 53.0
	Soft *N* = 28	9.11 (− 0.87, 19.1) + 16.5 (28.78) + 5.3 to + 27.6	0.72 0.005	− 43.3 LL to 61.52 UL − 39.9 to + 72.9
	Solid *N* = 28	5.88 (− 3.46, 15.22) + 8.6 (26.90) − 1.9 to + 19.0	0.21 0.1	− 43.16 LL to 54.92 UL − 44.1 to + 61.3
Degree of esophageal residue	Thin liquids *N* = 28	18.58 (11.76, 25.39) + 28.6 (22.97) + 19.7 to + 37.5	0.00 < 0.001	− 17.19 LL to 54.36 UL − 16.4 to + 73.6
	Puree *N* = 29	5.57 (− 72, 11.85) + 10.8 (3.73) + 3.2 to + 18.4	0.81 0.007	− 27.05 LL to 38.19 UL + 3.5 to + 18.1
	Soft *N* = 27	10.3 (2.32, 18.28) + 18.0 (21.96) + 9.3 to +26.7	0.13 < 0.001	− 31.6 to 52.2 UL − 25 to + 61.0
	Solid *N* = 29	7.93 (0.14, 15.72) + 10.0 (21.09) + 2.0 to + 18.0	0.046 0.02	32.95 (LL) to 48.81 UL − 31.3 to + 51.3

*Mean difference = mean visual analogue scale measurement for FEES – mean visual analogue scale measurement for VF. Min–Max = 0–100 with a higher score meaning more residue. A positive difference = a higher score from FEES; a negative difference = a higher score from VF

*LL* lower limit, *UL* upper limit, *VF* videofluoroscopy, *FEES* Fibreoptic Endoscopic Evaluation of Swallow

### AIM 3: Comparison of Features Using VF and FEES

#### Intra- and Inter-rater Reliability

Detailed results are contained in electronic supplementary material 3 and show the following:

Intra-rater reliability of free marginal kappa > 0.6 was achieved on 100% of categorical questions, (odd numbered questions on the rating scale—see electronic supplementary material 3). Inter-rater reliability for categorical data was less robust with free marginal kappa of > 0.6 achieved on 33% (4/12) of questions for videofluoroscopy and 42% (5/12) for FEES. Intra-rater reliability of ICC > 0.6 was achieved on 58% (7/12) of continuous questions (even numbered questions on the rating scale– see electronic supplementary material 3). Inter-rater reliability of ICC > 0.6 for continuous data was achieved on 25% (3/12) questions for videofluoroscopy and 33% (4/12) questions for FEES.

Given the majority of missing data under FEES, we excluded the neopharynx from the analysis in both instruments to give a direct comparison. Overall agreement is summarised in Table 4, using Fleiss kappa.

**Table 4:** Summary of agreement between 3 raters on 240 swallow sequences. For FEES, exclusions were recorded when one or more raters were unable to rate a sequence. (All swallows are tabulated)

	All − ( )	One + ( )	Two + ( )	Three + ( )	Excluded	Agreement
FEES	0	8	66	144	22	77% observed Kappa = 0.18
VF	3	43	67	127	0	69% observed Kappa = 0.10

Green indicates no residue, red indicates presence of residue

**Fig. 1:**
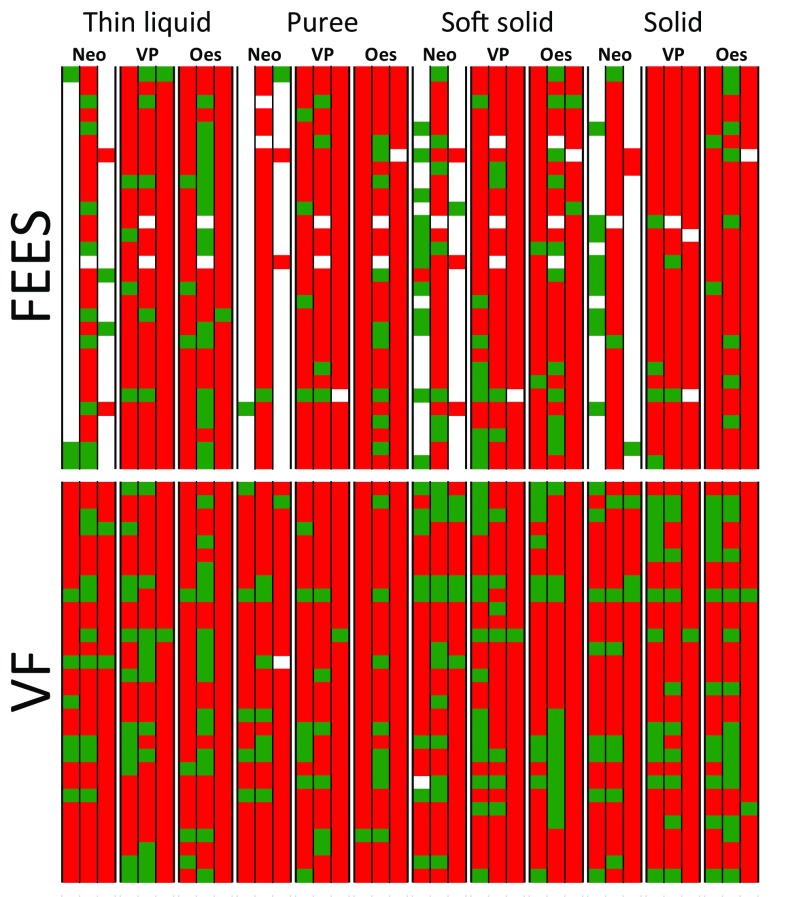
The results of simultaneous swallow assessments on 30 patients by two instruments (FEES and VF) and 3 expert raters. Residue was assessed for 4 food consistencies (thin liquid, puree, soft solid, solid) and on 3 anatomical structures (neopharynx, voice prosthesis, upper esophagus), by 3 raters. The columns represent these ratings. Each row represents one patient. A red square indicates ‘residue present’, and green indicates ‘no residue’. If the rater could not make a judgment, then the square is white. In total, there are 30 patients × 3 raters × 4 consistencies × 3 features = 1080 ratings for VF, and ratings of the same 1080 swallow sequences for FEES. The order of patients is the same for VF and for FEES. Therefore (for example), the top left square in the top and bottom panel relates to the same swallow sequence

Observed pairwise agreement was reasonably good, but there was heavy bias with about 80% of all ratings being positive (see Fig. 1). Consequently, the probability of agreement by chance is almost 70%; this maps to kappa = 0. We present the kappa statistic with some reservations, because it is considered to give a pessimistic view of reliability under these circumstances.

Primarily, the better agreement for FEES came from the 180 cases with full consensus that the sequence was abnormal. Raters scored more ‘normal’ sequences on VF and consensus on these was poor, albeit better for VF. This can be seen in Fig. 1 where agreement about the green boxes is clearly better for VF, though still poor. We also note that 22 sequences could not be rated on FEES.

### Inter-instrument Agreement

As earlier, we excluded the neopharynx from this assessment given that most of these swallow sequences were un-rateable on FEES. The results are shown in Table 5.

**Table 5:** Summary of agreement between VF and FEES on 218 swallow sequences where the three raters reached consensus on the outcome for both instruments

	VF − ( )	VF + ( )	Total
FEES + ( )	39	171	210
FEES − ( )	2	6	8
Total	41	177	218

The 22 excluded swallow sequences are the same as those recorded in Table 4. (Non excluded swallows tabulated only)

Green indicates no residue, red indicates presence of residue

Overall pairwise agreement was 173/218 swallow sequences, or 79% (kappa = − 0.03). The agreement between FEES and VF is exactly what one would expect by chance alone. This is evident from Fig. 1, where there is poor correspondence in green areas between the top and bottom panels.

Considering now the overall bias, there was a significant bias towards FEES scoring more positive findings (McNemar’s test, *P* < 0.001). This is indicated by the discordant pairs in Table 2, top-left and bottom-right. In 39/45 cases, the FEES scored the positive and the VF was negative (odds ratio = 6.5, 95% CI 2.7–18.8).

## Discussion

This study provides preliminary evidence for the presentation of dysphagia following laryngectomy. We assessed the patients using the same tools, methods and expert reviewers that manage dysphagia in the non-laryngectomy population.

### AIM 1: Presence of Residue in the Post Laryngectomy Swallow

The first objective of this study was to ascertain which dysphagia evaluation tool more accurately identified presence of residue in the neopharynx, on the voice prosthesis and in the upper esophagus. Presence of residue is important for laryngectomy patients because it may delay the swallow, may necessitate the need to alternate food with swallow to clear residue and causes patients to swallow more than once. Poor pharyngeal clearance post laryngectomy resulting in residue has previously been described [28, 14]. Videofluoroscopy provided greater identification than FEES on all consistencies in the neopharynx. It is possible that raters may have found it easier to identify this area on the broader field of view provided by videofluoroscopy X-ray image than on the surface anatomy view provided by FEES. Videofluoroscopy also scored more highly than FEES for the identification of puree residue on the voice prosthesis. This could be due to the propensity of the puree material (custard) to collect on the tip of the endoscope thereby obscuring the view on FEES but not on videofluoroscopy. The raters were therefore unable to rate puree on FEES because they were unable to see anything. The use of a less glutinous puree consistency may have reduced adhesion of puree to the tip of the endoscope. A significant limitation of this study is the presence of missing values as a result of the inability of raters to view residue particularly in the neopharyx. Identification of residue in the upper esophagus was similar on both tools except for solid for which FEES appeared to offer an advantage. Further research would be beneficial to ascertain whether this is an incidental finding or indicative of the difficulty inherent in coating a solid bolus with sufficient barium to ensure comprehensive identification of residue on videofluoroscopy. This study involved coating the solid biscuit bolus with a barium preparation. Utilising a biscuit baked with barium may have yielded a different result.

### AIM 2: Degree of Residue

The next objective of this study was to investigate degree of residue. Poor mucosal clearance resulting in residue has previously been described as a feature of post laryngectomy swallowing [14, 28]. The greater the degree of residue, the longer and more laborious mealtimes may become for patients. Videofluoroscopy scored higher for identifying degree of residue in the neopharynx. However, for the upper esophagus and on the voice prosthesis, videofluoroscopy and FEES scored similarly, with the exception of thin liquids on the voice prosthesis and thin liquids and solids in the esophagus. Thus, it would appear that for examining the degree of residue in the neopharynx VF is better, whilst for the voice prosthesis and upper esophagus both FEES and VF may be used.

Interestingly, a previous study [14] indicated that dysphagia was not self reported by some patients despite evidence of significant residue. It may be worth considering whether some degree of residue should be regarded as ‘normal’ post laryngectomy. If some residue is judged as normal in the post-laryngectomy swallow, then we must define ‘abnormality’ to identify how much residue constitutes normality.

If we consider residue of any amount to be abnormal, then on the evidence of this study we may need to offer every laryngectomy patient the opportunity of some intervention, such as strengthening tongue base retraction to promote bolus clearance through the reconstructed pharynx. However, we need tools with the specificity to more clearly delineate the nature of the underlying swallowing physiology causing dysphagia post laryngectomy.

### AIM 3: Comparison of Features Using VF and FEES

In order to explore which tool (VF or FEES) may be better for assessing laryngectomy swallow we had to rate the findings from these assessments. Interpretation of a swallowing image, whether elicited from videofluoroscopy or from FEES, is largely based on visual judgment and is inherently subjective in nature. The rating scale used in this study to measure expert raters judgment showed poor intra-rater reliability for FEES images, and the poor inter-rater reliability for both videofluoroscopy and FEES. Previous studies [50–52] have also identified poor inter-rater reliability on various parameters of videofluoroscopy swallow evaluation highlighting the subjective nature of these assessments. Free marginal kappa was used to evaluate reliability for categorical data. Free marginal kappa is approximately equivalent to Fleiss/Cohen kappa under best possible conditions where there are equal numbers of each category to be assigned. In the absence of best possible conditions, free marginal kappa is likely to be higher than Fleiss/Cohen kappa. In our study inter-rater reliability was worse for continuous data than for categorical data, where continuous data was derived from visual analogue scales (VAS) to indicate degree of residue. Previously VAS have been proposed as a more precise method of measuring residue compared to categorical scales [53, 54], but our data suggest that reliability is poor and so further research is required to find the best way to evaluate degree of residue. Bolus consistency has been identified as a factor affecting rater agreement levels on FEES [55] with lower agreement for thin liquid than for thick liquid. The impact of consistency on observer agreement remains underexplored and may require further investigation in relation to this study which utilised multiple consistencies. Part of the training for the expert raters in this study included group discussion and comparison of rated images and this may have improved inter-rater reliability because others [56] have indicated that levels of agreement are lowest when raters worked alone in judging videofluoroscopy.

In our group of patients, the summary reliability of the data as measured by the kappa statistic is poor. There are two likely explanations: first, we suspect this task has particular challenges for clinicians who may re-calibrate their internal reference to this group of patients to varying degrees. For example, consider FEES*, thin liquid, esophagus* in Fig. 1. One of the three raters scored 17 normal swallows whereas the other two scored three and one respectively. This suggests that one rater has a completely different internal reference as to what is ‘normal’ compared to the other two. One would expect that experts would have far better agreement. Secondly, the kappa statistic has known idiosyncrasies. We reported an observed agreement (i.e. the number of times when a pair of raters agreed) of around 80%. In a balanced task with equal numbers of positive and negative cases this would correspond to kappa = 0.6, considered subjectively ‘good’ agreement [57]. In our sample kappa is around 0.1. The proponents of kappa would point out that the context of the rating task is important. Here we are measuring a group of patients at one extreme (i.e. without a larynx). In this specific situation where there is relatively little variability between patients, the rating scale must have better resolution and accuracy. There is a direct analogy with other measuring instruments. A weighing scale that is designed for adult patients up to 150 kg would not be the right tool to measure neonates who are all in the range of 2–10 kg. We need a more specific tool in this patient group.

The agreement between VF and FEES was even worse, and indeed was no better than chance (kappa = − 0.03). The statistical interpretation of this finding is worth exploring. If one picked any two swallow ratings *completely at random*, you would expect those ratings to agree about 80% of the time, purely by chance. We observed 79% agreement between VF and FEES. This is slightly worse even than chance would predict, so kappa was slightly negative. Since we do not have a gold standard in this study, and since neither instrument showed a relationship with self-reported swallow problems, we cannot say which, if any, instrument has clinical value. Nevertheless, we report significantly more positive findings on FEES. This is in keeping with a previous study, [47] where using a 4-point residue scale there was a consistent difference of about 1 point between FEES (higher) and VF (lower) using simultaneous measurement. As with Kelly’s work, without a gold standard it is not possible to say which is correct. FEES is the more sensitive tool, but may in some circumstances be detecting a thin coating of residue that is clinically unimportant i.e. a false positive.

## Conclusion

This study has demonstrated that dysphagia is an issue post laryngectomy with residue a significant symptom as measured by instrumental evaluation. However, this study has also highlighted the issues with rater reliability in both identifying presence and degree of residue. As a consequence of the low aspiration risk presented post laryngectomy, the areas of both dysphagia evaluation and intervention have remained largely under explored in this population. While both videofluoroscopy and FEES may be beneficial for evaluating aspects of post laryngectomy swallowing, further research is required to optimize the use of these and alternative tools in this patient cohort. The ability to identify symptoms of dysphagia using evaluation tools with established reliability is likely to become increasing important to enable appropriate interventions to be developed for this sub group of head and neck cancer patients.

## Electronic Supplementary Material

Below is the link to the electronic supplementary material.

Supplementary material 1 (DOCX 110 kb)

Supplementary material 2 (DOCX 62 kb)

Supplementary material 3 (DOCX 103 kb)
