# Supplementary material for: An Investigation of the Post-laryngectomy Swallow Using Videofluoroscopy and Fiberoptic Endoscopic Evaluation of Swallowing (FEES)
Source: Dysphagia. 2018 Jan 19;33(3):369–79. doi: 10.1007/s00455-017-9862-7 (PMC5958146; doi:10.1007/s00455-017-9862-7)
Supplement: Supplementary file 1 — Supplementary material 1 (DOCX 110 kb) [file 455_2017_9862_MOESM1_ESM.docx]

**An investigation of the post-laryngectomy swallow using Videofluoroscopy and Fiberoptic Endoscopic Evaluation of Swallowing (FEES). *Dysphagia.***

**Dysphagia Visual Analogue Scale (VAS) Chart**

**Image No: _________________________________________________________**

**Raters initials: ____________________________________________________________**

**Residue**

**Q1. Is there neopharyngeal residue on thin liquids? Yes *If yes, rate Q10b below*  No *If no, rate Q11a***

**Q2. How much neopharyngeal residue is there on thin liquids?**

**Minimal**

**Severe**

**Q3. Is there voice prosthesis residue on thin liquids? Yes *If yes, rate Q11b below***

**No *If no, go to Q12a***

**Q4. How much voice prosthesis residue is there on thin liquids?**

**Minimal**

**Severe**

**Q5. Is there oesophageal residue on thin liquid? Yes *If yes, rate Q12b below***

**No**

**Q6. How much oesophageal residue is there on thin liquids?**

**Minimal**

**Severe**

**Q7 Is there neopharyngeal residue on puree? Yes *If yes, rate Q13b below***

**No *If no, go to Q14a***

**Q8. How much neopharyngeal residue is there on puree?**

**Severe**

**Minimal**

**Q9. Is there voice prosthesis residue on puree? *Yes If yes, rate Q14b below***

***No If no, go to Q15a***

**Q10. How much voice prosthesis residue is there on puree?**

**Severe**

**Minimal**

**Q11. Is there oesophageal residue on puree?  *Yes If yes, rate Q15b below***

***No If no, go to Q16a***

**Q12. How much oesophageal residue is there on puree?**

**Severe**

**Minimal**

**Q13. Is neopharyngeal residue visible on soft? Yes *If yes, rate Q16b below***

**No *If no, go to Q17a***

**Q14.** **How much neopharyngeal residue is there on soft?**

**Minimal**

**Severe**

**Q15. Is there voice prosthesis residue on soft? Yes *If yes, rate Q17b below***

**No *If no, go to Q18a***

**Q16. How much residue is on the voice prosthesis on soft?**

**Severe**

**Minimal**

**Q17. Is there oesophageal residue on soft? Yes *If yes, rate Q18a & b below***

**No *If no, go to Q19***

**Q18. How much oesophageal residue is there on soft?**

**Minimal**

**Severe**

**Q19. Is there neopharyngeal residue on solid? Yes *If yes, rate Q19b below***

**No *If no, go to Q20a***

**Q20 How much neopharyngeal residue is there on solid?**

**Severe**

**Minimal**

Q21. **Is there voice prosthesis residue on solid?** Yes ***If yes, rate Q20b below***

**No *If no, go to Q21a***

**Q22. How much voice prosthesis residue is there on solid?**

**Severe**

**Minimal**

**Q23 Is there oesophageal residue on solid? Yes *If yes, rate Q22b below***

**No *If no, go to Q25***

**Q24 How much oesophageal residue is there on solid?**

**Severe**

**Minimal**

**Other observations/comments**

**Please continue on another sheet if you have further comments.**

**Thank you for completing this questionnaire!**
