# Supplementary material for: An Investigation of the Post-laryngectomy Swallow Using Videofluoroscopy and Fiberoptic Endoscopic Evaluation of Swallowing (FEES)
Source: Dysphagia. 2018 Jan 19;33(3):369–79. doi: 10.1007/s00455-017-9862-7 (PMC5958146; doi:10.1007/s00455-017-9862-7)

**An investigation of the post-laryngectomy swallow using Videofluoroscopy and Fiberoptic Endoscopic Evaluation of Swallowing (FEES). *Dysphagia.***

**Fig 1. Bland and Altman plots, missing values removed**


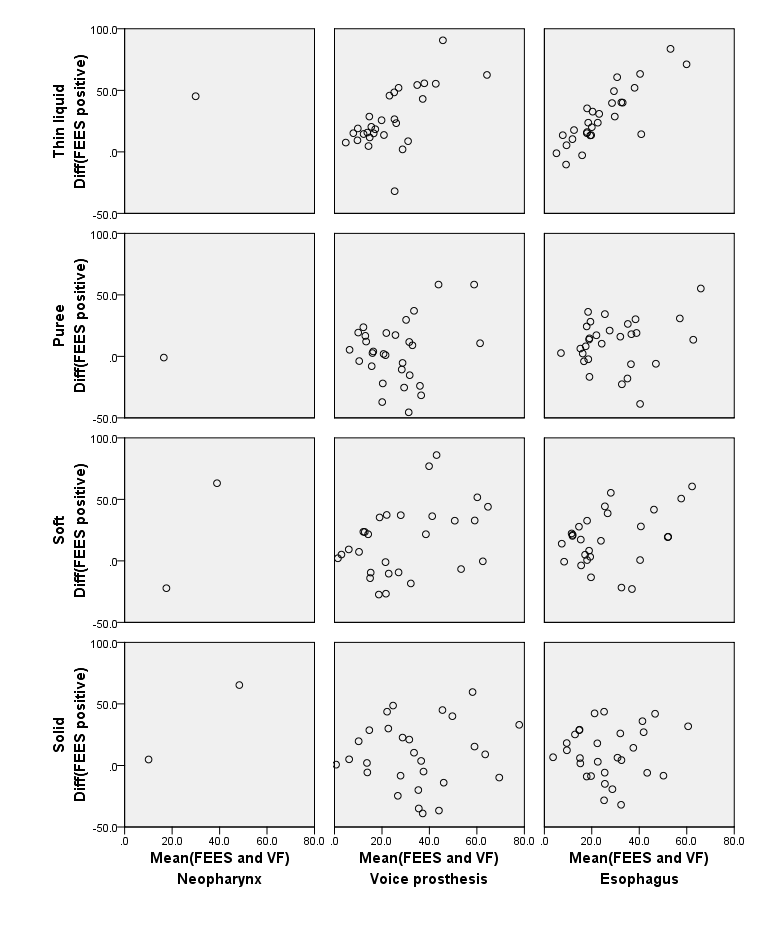

Supplement: Supplementary file 2 — Supplementary material 2 (DOCX 62 kb) [file 455_2017_9862_MOESM2_ESM.docx]
