# Supplementary material for: An Investigation of the Post-laryngectomy Swallow Using Videofluoroscopy and Fiberoptic Endoscopic Evaluation of Swallowing (FEES)
Source: Dysphagia. 2018 Jan 19;33(3):369–79. doi: 10.1007/s00455-017-9862-7 (PMC5958146; doi:10.1007/s00455-017-9862-7)
Supplement: Supplementary file 3 — Supplementary material 3 (DOCX 103 kb) [file 455_2017_9862_MOESM3_ESM.docx]

**An investigation of the post-laryngectomy swallow using Videofluoroscopy and Fiberoptic Endoscopic Evaluation of Swallowing (FEES). *Dysphagia.***

| Question | VF rater 1 | FEES rater 1 | VF rater 2 | FEES rater 2 | VF rater 3 | FEES rater 3 |
| --- | --- | --- | --- | --- | --- | --- |
| Are there secretions present | 0.7 | 1.0 | 1.0 | 1.0 | 1.0 | 1.0 |
| Is there a pseudoepiglottis present | 1.0 | 1.0 | 1.0 | 1.0 | 1.0 | 0.4 |
| Is the voice prosthesis visible | No variance | 1.0 | 1.0 | 1.0 | 1.0 | 1.0 |
| Is there neopharyngeal residue on thin liquids | 1.0 | 1.0 | 1.0 | 0.4 | 1.0 | 1.0 |
| Is there voice prosthesis residue  on thin liquids | 1.0 | 1.0 | 1.0 | 1.0 | 1.0 | 1.0 |
| Is there oesophageal residue on thin liquids | 1.0 | 1.0 | 1.0 | 1.0 | 1.0 | 1.0 |
| Is there neopharyngeal residue on puree | 1.0 | 1.0 | 1.0 | 1.0 | 1.0 | 1.0 |
| Is there voice prosthesis residue on on puree | 1.0 | 1.0 | 1.0 | 1.0 | 1.0 | 1.0 |
| Is there oesophageal residue on puree | 1.0 | 1.0 | 1.0 | 1.0 | 1.0 | 0.7 |
| Is there neopharyngeal residue on soft | 0.7 | 1.0 | 1.0 | 0.1 | 1.0 | 1.0 |
| Is there voice prosthesis residue on soft | 1.0 | 1.0 | 1.0 | 1.0 | 1.0 | 1.0 |
| Is there oesophageal residue on soft | 1.0 | 1.0 | 1.0 | 1.0 | 1.0 | 0.7 |
| Is there neopharyngeal residue on solid | 1.0 | 0.7 | 1.0 | 1.0 | 1.0 | 0.7 |
| Is there voice prosthesis residue on solid | 1.0 | 0.7 | 1.0 | 1.0 | 0.7 | 1.0 |
| Is there oesophageal residue on solid | 1.0 | 1.0 | 1.0 | 1.0 | 0.7 | 0.7 |

**Table 6. Intra rater reliability – Categorical (Kappa)**

| Question | VF 1^st^ rating | VF 2^nd^ rating | FEES 1^st^ rating | FEES 2^nd^ rating |
| --- | --- | --- | --- | --- |
| Are there secretions present | 0.79 | 0.99 | 0.99 | 0.99 |
| Is there a pseudoepiglottis present | 0.79 | 0.79 | 0.60 | 0.60 |
| Is the voice prosthesis visible | 0.60 | 0.60 | 0.99 | 0.99 |
| Is there neopharyngeal residue on thin liquids | 0.60 | 0.60 | -0.1 | -0.3 |
| Is there voice prosthesis residue  on thin liquids | 0.39 | 0.39 | 0.99 | 0.99 |
| Is there oesophageal residue on thin liquids | 0 kappa (no agreement) | 0 kappa (no agreement) | 0.19 | 0.19 |
| Is there neopharyngeal residue on puree | 0.60 | 0.60 | -0.1 | -0.39 |
| Is there voice prosthesis residue on on puree | 0.39 | 0.39 | 0.79 | 0.79 |
| Is there oesophageal residue on puree | 0.19 | 0.19 | 0.49 | 0.60 |
| Is there neopharyngeal residue on soft | 0.79 | 0.79 | -0.10 | -0.30 |
| Is there voice prosthesis residue on soft | 0.39 | 0.39 | 0.60 | 0.60 |
| Is there oesophageal residue on soft | 0.39 | 0.46 | 0.70 | 0.70 |
| Is there neopharyngeal residue on solid | 0.79 | 0.79 | -0.30 | -0.30 |
| Is there voice prosthesis residue on solid | 0.19 | 0.19 | 0.99 | 0.99 |
| Is there oesophageal residue on solid | 0 | 0.19 | 0.30 | 0.39 |

**Table 7. Inter rater reliability – Categorical (Kappa)**

| Question | VF rater 1 | FEES rater 1 | VF rater 2 | FEES rater 2 | VF rater 3 | FEES rater 3 |
| --- | --- | --- | --- | --- | --- | --- |
| **Rate degree of neopharyngeal residue on thin liquids** | 0.94 | 0.91 | 0.82 | No variance | 0.90 | 0.99 |
| **Rate degree of VP residue on thin liquids** | 0.93 | 0.96 | 1.0 | 0.84 | -0.32 | 0.88 |
| **Rate degree of oesophageal residue on thin liquids** | No variance | 0.96 | 0.67 | 0.97 | 0.57 | 0.56 |
| **Rate degree of neopharyngeal residue on puree** | 0.92 | 0.63 | 0.97 | No variance | 0.95 | 0.89 |
| **Rate degree of VP residue on puree** | 0.2 | 0.22 | 0.88 | 0.94 | 0.59 | 0.26 |
| **Rate degree of oesophageal residue on puree** | 1.0 | 0.65 | 0.66 | 0.67 | 0.84 | 0.13 |
| **Rate degree of neopharyngeal residue on soft** | 0.98 | 0.96 | 0.95 | No variance | 0.95 | 0.98 |
| **Rate degree of VP residue on soft** | 0.96 | 0.99 | 0.99 | 0.98 | -0.23 | 0.88 |
| **Rate degree of oesophageal residue on soft** | 0.95 | 0.80 | 0.95 | 0.97 | 0.90 | -0.09 |
| **Rate degree of neopharyngeal residue on solid** | 0.87 | 0.93 | 0.95 | No variance | 0.93 | 0.96 |
| **Rate degree of residue on VP on solid** | 0.56 | 0.99 | 0.96 | 0.88 | 0.58 | 0.97 |
| **Rate degree of oesophageal residue on solid** | No variance | 0.93 | 0.90 | 0.99 | 0.82 | 0.87 |

**Table 8. Intra rater reliability – Continuous (ICC)**

| Question | VF 1^st^ rating | VF 2^nd^ rating | FEES 1^st^ rating | FEES 2^nd^ rating |
| --- | --- | --- | --- | --- |
| **Rate degree of neopharyngeal residue on thin liquids** | 0.49 | 0.55 | 0.64 | 0.36 |
| **Rate degree of VP residue on thin liquids** | 0.77 | 0.21 | 0.83 | 0.72 |
| **Rate degree of oesophageal residue on thin liquids** | 0.19 | 0.28 | 0.35 | 0.15 |
| **Rate degree of neopharyngeal residue on puree** | 0.87 | 0.83 | -0.25 | 0.20 |
| **Rate degree of VP residue on puree** | 0.44 | 0.23 | 0.10 | 0.35 |
| **Rate degree of oesophageal residue on puree** | 0.14 | 0.25 | 0.31 | 0.47 |
| **Rate degree of neopharyngeal residue on soft** | 0.91 | 0.91 | 0.70 | 0.70 |
| **Rate degree of oesophageal residue on soft** | 0.26 | 0.47 | -0.06 | 0.08 |
| **Rate degree of neopharyngeal residue on solid** | 0.94 | 0.92 | 0.66 | 0.64 |
| **Rate degree of residue on VP on solid** | 0.18 | 0.73 | 0.93 | 0.88 |
| **Rate degree of oesophageal residue on solid** | 0.25 | 0.25 | 0.12 | 0.18 |

**Table 9. Inter rater reliability – Continuous (ICC)**
